# Influence of Dystrophin Isoform Deficiency on Motor Development in Duchenne Muscular Dystrophy

**DOI:** 10.1002/acn3.70097

**Published:** 2025-06-24

**Authors:** Mary Chesshyre, Deborah Ridout, Georgia Stimpson, Laurent Servais, Giovanni Baranello, Adnan Manzur, UK NorthStar Clinical Network, M. Scoto, A. Sarkozy, P. Munot, S. Robb, E. Chan, V. Robinson, W. Girshab, V. Crook, E. Milev, L. Abbott, A. Wolfe, E. O’Reilly, J. Watts‐Whent, N. Burnett, R. Thomas, R. Terespolsky, O. Martinaeu, J. Longatto, V. Straub, C. Bettolo, M. Guglieri, J. Diaz‐Manera, G. Tasca, M. Elseed, R. Muni‐Lofra, M. James, D. Moat, J. Sodhi, K. Wong, E. Robinson, E. Groves, R. Rabb, D. Parasuraman, H. McMurchie, H. Chase, Tracey Willis, C. Rylance, N. Birchall, E. Wright, A. Childs, K. Pysden, C. Martos, D. Roberts, L. Pallant, S. Walker, A. Henderson, R. Madhu, R. Karuvattil, Y. Balla, S. Gregson, S. Clark, E. Wraige, H. Jungbluth, V. Gowda, M. Vanegas, J. Sheehan, A. Schofield, C. Smith, I. Hughes, E. Whitehouse, S. Warner, E. Reading, N. Emery, J. Moustoukas, K. Strachan, M. Ong, M. Atherton, N. Mills, S. Sanchez Marco, A. Saxena, K. Skone, J. TeWaterNaude, H. Davis, C. Wood, A. Majumdar, A. Murugan, I. Guarino, R. Tomlinson, H. Jarvis, L. Wills, C. Frimpong, J. Watson, G. Cobb, G. Robertson, P. Brink, J. Burslem, C. Adams, J. Wong, S. Joseph, I. Horrocks, J. Dunne, M. DiMarco, S. Brown, S. McKenzie, K. Torne, R. Mohamed, V. Velmurugan, M. Prasad, S. Sedehizadeh, A. Schugal, R. Keetley, S. Williamson, K. Payne, E. Dowling, P. Fenty, C. de Goede, A. Parkes, K. Baxter, M. Illingworth, N. Bhangu, S. Geary, J. Palmer, K. Shill, S. Tirupathi, A. Shah, D. O’Donogue, J. McVeigh, J. McFetridge, G. Nicfhirleinn, H. Beattie, T. Leyland, K. Stevenson, N. Hussain, D. Baskaran, Z. Lambat, R. Sullivan, L. Locke, G. Ambegaonkar, D. Krishnakumar, J. Taylor, J. Moores, E. Stephen, J. Tewnion, S. Ramdas, M. Sa, A. Skippen, M. Khries, C. Lilien, H. Ramjattan, F. Taylor, H. English, K. Stewart, F. Flint, E. Bartram, R. Noble, Francesco Muntoni

**Affiliations:** ^1^ Dubowitz Neuromuscular Centre UCL Great Ormond Street Institute of Child Health London UK; ^2^ Population, Policy and Practice Research and Teaching Department UCL Great Ormond Street Institute of Child Health London UK; ^3^ MDUK Oxford Neuromuscular Centre, STRONG Team Office Level 3, Academic Centre, John Radcliffe Oxford UK; ^4^ NIHR Oxford Biomedical Research Centre John Radcliffe Hospital Oxford UK; ^5^ Neuromuscular Center, Department of Paediatrics University of Liege, University Hospital of Liege Liege Belgium; ^6^ NIHR Great Ormond Street Hospital Biomedical Research Centre UCL Great Ormond Street Institute of Child Health London UK

**Keywords:** Duchenne muscular dystrophy, dystrophin isoform, motor development, north star ambulatory assessment

## Abstract

**Objective:**

In Duchenne muscular dystrophy (DMD), lack of the shorter dystrophin isoforms Dp140 and Dp71 is associated with increased central nervous system (CNS) involvement. We aimed to investigate how CNS involvement affects motor development in young DMD boys.

**Method:**

Three hundred and forty‐two DMD boys aged 3–6 years were subdivided according to *DMD* mutation expected effects on isoform expression: Group 1 (Dp427 absent, Dp140/Dp71 present, *n* = 170); Group 2 (Dp427/Dp140 absent, Dp71 present, *n* = 154) and Group 3 (Dp427/Dp140/Dp71 absent, *n* = 18). Mixed effects logistic regression was used to investigate relationships between isoform group and the odds of achieving higher North Star Ambulatory Assessment (NSAA) subitem scores for 15 subitems, adjusting for age at visit and glucocorticoid exposure.

**Results:**

The odds of achieving a full score of 2 were significantly lower for 11 NSAA subitems in Group 2 compared to Group 1, 5 NSAA subitems in Group 3 compared to Group 1, and 2 NSAA subitems in Group 3 compared to Group 2. The odds of achieving a score of 2 or 1 compared to 0 were significantly lower in Group 2 compared to Group 1 for the 2 NSAA subitems studied using this comparison.

**Interpretation:**

We found strong and significant associations between the odds of achieving higher NSAA subitem scores and expected patterns of dystrophin isoform involvement, with a cumulative effect of loss of isoforms. This suggests an important relationship between dystrophin isoforms in the brain and the ability to carry out gross motor milestones.

## 1 Introduction

Duchenne muscular dystrophy (DMD) is caused by *DMD* mutations leading to a complete or almost complete absence of the dystrophin protein [1]. The dystrophin protein exists in several different isoforms, driven by internal promoters located along the *DMD* gene [2]. Dp427_m_ is the predominant full‐length isoform expressed in muscle; the same isoform is also expressed in brain together with the more abundant Dp427_c_ expressed in the human brain [3]. Two shorter isoforms, Dp140 and Dp71, encoded by unique promoters located further downstream compared to the Dp427 promoter, are expressed at high levels in the adult human brain and the lack of Dp140 and Dp71 has been found to be associated with higher rates of DMD central nervous system involvement (CNS), including intellectual disability and other neurodevelopmental comorbidities [3, 4, 5, 6, 7].

The North Star Ambulatory Assessment (NSAA) is a 17‐item DMD‐specific motor function scale. It is an ordinal scale with a maximum possible total score of 34 [8]. Each item is scored 0 (unable to achieve independently), 1 (achieves goal independent of physical assistance from another with a modified method) and 2 (‘Normal’, achieves goal independent of physical assistance from another with no obvious modification of activity).

In this study, we hypothesised that young DMD boys with *DMD* mutations located in areas of the gene predicted to affect Dp140 or Dp140 and Dp71 would display deficits in the ability to acquire motor developmental milestones, as assessed by the achievement of higher scores in the subitems of the NSAA, with a cumulative effect of loss of isoforms. To investigate this, we evaluated the relationship between dystrophin isoform group and the odds of achieving higher scores in NSAA subitems, adjusting for age at visit and glucocorticoid (GC) exposure.

## 2 Methods

### 2.1 Data Sources

Clinical data was gathered from the AFM funded ‘Outcome Measures in DMD: A Natural History Study’ (NCT 02780492) and the UK NorthStar Clinical Network.

The UK NorthStar Clinical Network is a clinical network of neuromuscular centres looking after children and adults with DMD in the United Kingdom coordinated from the Dubowitz Neuromuscular Centre in London [9]. The collected data is stored in an electronic database managed by CertusLtd [9]. Standard operating procedures for assessments are used, and there is a national training programme for centres. Data is collected prospectively at routine clinic appointments. Participants were recruited from this network. Written informed consent was obtained for the collection of clinical data, and the UK NorthStar Clinical Network project has Caldicott Guardian approval. All clinical assessments are conducted according to the principles of the Declaration of Helsinki (2000) and its later amendments and the Principles of Good Clinical Practice.

The AFM funded study was an international, multicentre, prospective, longitudinal, natural history study involving five European centres: London (UCL Great Ormond Street Institute of Child Health), Newcastle (John Walton Muscular Dystrophy Research Centre), Paris (Institute of Myology), Leiden (Leiden University Medical Centre) and Nijmegen (Radboud University Medical Centre). The study protocol, consent and assent documents were approved by the ethical review boards at participating institutions. Written informed consent was obtained from study participants and/or their parent/legal guardian.

### 2.2 Study Design and Participants

Three hundred and forty‐two participants with DMD aged from 3.0 years to under 6.5 years were included. In this manuscript, we consider a ‘full score’ to be a score of 2 for an NSAA subitem. NSAA assessments where 6 or more NSAA subitem scores were missing were excluded. Participants were grouped into 2 GC groups. The ‘GC naïve’ group consisted of those who were not on GC for 6 months or more at the first visit and the ‘GC treated group’ consisted of those who were on GC for 6 months or more at the first visit.

### 2.3 *DMD* Mutation Data

Included participants had a *DMD* mutation that was predicted to be out of frame and/or frameshift and/or a nonsense mutation. Participants were grouped into 3 isoform groups based on predicted *DMD* mutation effects on dystrophin isoform expression: Group 1 (Dp427 absent, Dp140/Dp71 present), Group 2 (Dp427/Dp140 absent, Dp71 present). Group 1 consisted of participants with *DMD* mutations only involving the genomic region upstream of intron 44; Group 2 consisted of participants with *DMD* mutations involving the region from exon 51 to exon 62 inclusive, and not the region of exon 63 or downstream of exon 63; and Group 3 consisted of participants with *DMD* mutations involving exon 63 and/or the region downstream of exon 63 [10]. Participants with *DMD* mutations involving exons 45 to 50 inclusive and not involving the genomic region of exon 51 and/or downstream of exon 51 were excluded from the dystrophin isoform cohorts, as it is difficult to predict the effects of these mutations on Dp140 expression, as the Dp140 promoter is located in intron 44, and the stretch of exons between 45 and 50 are part of the 5′ untranslated region of this isoform [10, 11].

### 2.4 Statistical Analysis

Mixed effects logistic regression was used to investigate relationships between isoform group and the odds of achieving higher NSAA subitem scores for a total of 15 NSAA subitems, adjusting for age at visit and GC group. Mixed effects logistic regression was used to explore the relationships between isoform group and the odds of achieving a score of 2 compared to a score of 0 or 1 for 13 of the 17 NSAA subitems, adjusting for age at visit and GC group. These models accounted for the longitudinal data structure. For hop right leg and hop left leg, only 1 and 2 participants in Group 3 achieved a score of 2, respectively. Therefore, for 2 of the NSAA subitems, hop right leg and hop left leg, mixed effects logistic regression was used to explore the relationships between isoform group and the odds of achieving a score of 2 or 1 compared to a score of 0, adjusting for age at visit and GC group. The NSAA subitems stand and walk were not included in the analysis due to the high numbers of participants achieving a score of 2 for these subitems. For outcomes where the relationship with age was not linear, we fitted piecewise linear splines and, based on visual inspection of the data, chose knot points at ages 4 or 5 years.

In order to better understand the clinical relevance of our findings, the predicted percentage of boys achieving a full score of 2 (for 13 subitems) or a score of 2 or 1 (for hop right leg and hop left leg) was derived for those in the GC naïve group from the mixed effects logistic regression models. Bonferroni correction for 3 isoform groups was applied, resulting in a *p* value significance threshold of 0.017. Statistical analysis and creation of figures were conducted in R studio version 4.2.2 (2022‐10‐31) and packages lme4 and MASS were used to carry out logistic regression.

Previous studies have reported a milder phenotype in DMD boys with a *DMD* mutation that is exon 44 skippable [12, 13, 14, 15] or a deletion of exons 3 to 7 [12, 15]. A sensitivity analysis was conducted excluding those participants with a *DMD* mutation expected to be exon 44 skippable or a deletion of exons 3 to 7.

## 3 Results

### 3.1 Participant Characteristics

Overall, 342 participants were included, of whom 170 were in Group 1, 154 were in Group 2 and 18 were in Group 3 (Table 1). 899 visits were included, and participants ranged in age from 3.00 to 6.49 years. Data was included from the UK NorthStar Clinical Network only for 338 participants, from the AFM funded study only for three participants, and from both the UK NorthStar Clinical Network and the AFM funded study for one participant. Please see the Appendix 1 for additional information about participants' GC medication and regime.

**TABLE 1:** Patient characteristics.

Characteristic	Overall	Isoform group		
		Group 1	Group 2	Group 3
Total number of participants (*n*)	342	170	154	18
Age range (y)	3.00, 6.49	3.14, 6.48	3.00, 6.49	3.03, 6.48
Total number of visits (*n*)	899	433	413	53
Mean (SD) age at first visit (y)	4.96 (0.86)	5.04 (0.86)	4.91 (0.85)	4.57 (0.80)
Number in GC naïve group (*n* and %)	276 (80.70%)	143 (84.12%)	115 (74.68%)	18 (100%)
Mean (SD) age at last visit (y)	5.91 (0.60)	5.90 (0.61)	5.93 (0.58)	5.89 (0.66)

*Note:* Numbers are reported to two decimal places.

Abbreviations: %, percentage; GC, glucocorticoids; *n*, number; SD, standard deviation; y, years.

### 3.2 Interpretation of Odds and Odds Ratios

The odds of an event happening are defined as the probability that an event will occur, expressed as a proportion of the probability that the event will not occur [16]. The odds ratio (OR) is the odds of an event happening in one group expressed as a proportion of the odds of an event happening in another group [16]. The closer the OR is to 1, the smaller the difference in effect between the two groups [16]. If the OR is greater (or less) than 1, then the effects of being in one group are more (or less) than those of being in the other group [16].

Using stand on one leg right as an example (Table 2), an increase of 1 year in age was associated with an increase of 231%, (3.31–1)*100 = 231, in the odds of achieving a full score for stand on one leg right for those aged 3 to 6 years after adjusting for isoform group and GC group (95% CI 138%, 360%). Those in Group 2 had a reduction of 64%, (1–0.36)*100 = 64, in the odds of achieving a full score for stand on one leg right compared to those in Group 1, after adjusting for age at visit and GC group (95% CI 33%, 81%).

**TABLE 2:** The odds of achieving a full score of 2 compared to a score of 0 or 1 in 13 NSAA subitems according to age at visit, isoform group and GC group.

Subitem	Age at visit			Group 2 compared to Group 1		Group 3 compared to Group 1		Group 3 compared to Group 2		GC treated group compared to GC naïve group	
	Relevant age (y)	OR (95% CI)	*p*	OR (95% CI)	*p*	OR (95% CI)	*p*	OR (95% CI)	*p*	OR (95% CI)	*p*
Stand from chair	3 to 6	0.80 (0.62, 1.04)	0.102	0.43 (0.23, 0.78)	**0.006**	0.28 (0.08, 0.98)	0.046	0.66 (0.19, 2.27)	0.511	1.98 (0.87, 4.51)	0.101
Stand on one leg—right	3 to 6	3.31 (2.38, 4.60)	**< 0.001**	0.36 (0.19, 0.67)	**0.001**	0.02 (0.004, 0.15)	**< 0.001**	0.06 (0.01, 0.40)	**0.003**	1.20 (0.54, 2.64)	0.651
Stand on one leg—left	3 to 6	3.68 (2.27, 5.95)	**< 0.001**	0.20 (0.09, 0.42)	**< 0.001**	0.02 (0.002, 0.11)	**< 0.001**	0.08 (0.01, 0.52)	**0.008**	1.69 (0.69, 4.18)	0.252
Climb box step—right	3 to 4	8.20 (4.12, 16.32)	**< 0.001**	0.44 (0.24, 0.83)	**0.011**	0.23 (0.06, 0.86)	0.029	0.51 (0.13, 1.93)	0.321	1.51 (0.67, 3.41)	0.321
	5 to 6	1.44 (0.44, 4.65)	0.547								
Descend box step—right	3 to 4	6.95 (3.41, 14.15)	**< 0.001**	0.42 (0.24, 0.76)	**0.004**	0.23 (0.06, 0.87)	0.030	0.55 (0.15, 2.06)	0.378	1.12 (0.52, 2.40)	0.768
	5 to 6	1.10 (0.33, 3.68)	0.880								
Climb box step—left	3 to 4	5.32 (2.73, 10.36)	**< 0.001**	0.27 (0.14, 0.54)	**< 0.001**	0.17 (0.04, 0.74)	0.018	0.63 (0.15, 2.67)	0.533	1.59 (0.67, 3.76)	0.295
	5 to 6	1.90 (0.60, 6.03)	0.277								
Descend box step—left	3 to 6	2.22 (1.70, 2.91)	**< 0.001**	0.38 (0.21, 0.68)	**0.001**	0.21 (0.06, 0.73)	**0.015**	0.55 (0.16, 1.95)	0.355	1.03 (0.49, 2.16)	0.935
Lifts head	3	0.08 (0.02, 0.42)	**0.003**	0.35 (0.19, 0.63)	**< 0.001**	0.24 (0.06, 0.93)	0.039	0.69 (0.18, 2.66)	0.587	1.62 (0.76, 3.47)	0.210
	4 to 6	2.50 (0.22, 28.31)	0.460								
Gets to sitting	3 to 6	1.68 (1.17, 2.43)	**0.005**	0.59 (0.33, 1.06)	0.079	0.25 (0.06, 1.03)	0.054	0.42 (0.09, 1.89)	0.257	2.14 (1.03, 4.43)	0.040
Rise from floor	3 to 6	1.82 (1.10, 3.01)	0.020	0.49 (0.21, 1.15)	0.102	0.71 (0.12, 4.29)	0.708	1.44 (0.23, 8.93)	0.696	1.70 (0.59, 4.95)	0.327
Stands on heels	3 to 4	2.70 (1.33, 5.48)	**0.006**	0.36 (0.20, 0.65)	**0.001**	0.31 (0.08, 1.16)	0.082	0.86 (0.23, 3.26)	0.821	1.60 (0.76, 3.36)	0.217
	5 to 6	0.94 (0.28, 3.20)	0.923								
Jump	3 to 4	17.92 (7.22, 44.48)	**< 0.001**	0.23 (0.10, 0.53)	**0.001**	0.05 (0.01, 0.33)	**0.002**	0.22 (0.04, 1.38)	0.106	2.65 (0.93, 7.51)	0.068
	5 to 6	1.89 (0.43, 8.28)	0.396								
Walk run (10 m)	3 to 6	1.91 (1.28, 2.85)	**0.001**	0.18 (0.08, 0.45)	**< 0.001**	0.02 (0.002, 0.14)	**< 0.001**	0.10 (0.02, 0.69)	0.020	2.55 (0.88, 7.37)	0.084

*Note:* ORs for age at visit are adjusted for isoform group and GC group, ORs for isoform group comparisons are adjusted for age at visit and GC group, ORs for GC group are adjusted for age at visit and isoform group. ORs and 95% CIs are reported to two decimal places or to three decimal places when < 0.01. *p* values < 0.017 are highlighted in bold. *p* values are reported to three decimal places or < 0.001. For lifts head, a knot at age 4 was included in the model to allow for the OR for age at visit to be different for ages 3 and 4 to 6. For climb right, descend right, climb left, stand on heels and jump; a knot at age 5 was included in the model to allow for the OR for age at visit to be different for ages 3 to 4 and 5 to 6.

Abbreviations: CI, confidence interval; GC, glucocorticoids; OR, odds ratio; y, years.

### 3.3 Relationships Between Age at Visit and the Odds of Achieving Higher NSAA Subitem Scores

An increase of 1 year in age was associated with a significant increase in the odds of achieving a full score by 68% or more for those aged 3 to 6 for the following 5 subitems: stand on one leg—right, stand on one leg—left, descend box step—left, sit and walk run (10 m) (Table 2). An increase of 1 year in age was associated with a significant increase in the odds of achieving a full score by 170% or more for those aged 3 to 4 for the following 5 subitems: climb box step—right, descend box step—right, climb box step—left, stands on heels and jump. An increase of 1 year in age was associated with a significant decrease in the odds of achieving a full score by 92% for those aged 3 for lifts head.

An increase of 1 year in age was associated with a significant increase in the odds of achieving a score of 2 or 1 compared to a score of 0 by 410% or more for those aged 3 to 6 for hop right leg and hop left leg (Table 3).

**TABLE 3:** The odds of achieving a score of 2 or 1 compared to a score of 0 in hop right leg and hop left leg according to age at visit, isoform group and GC group.

Subitem	Age at visit		Group 2 compared to Group 1		Group 3 compared to Group 1		Group 3 compared to Group 2		GC treated group compared to GC naïve group	
	OR (95% CI)	*p*	OR (95% CI)	*p*	OR (95% CI)	*p*	OR (95% CI)	*p*	OR (95% CI)	*p*
Hop right leg	5.37 (3.01, 9.58)	**< 0.001**	0.25 (0.12, 0.53)	**< 0.001**	0.20 (0.04, 0.95)	0.043	0.79 (0.17, 3.74)	0.768	2.78 (1.16, 6.67)	0.022
Hop left leg	5.10 (2.96, 8.81)	**< 0.001**	0.27 (0.13, 0.56)	**< 0.001**	0.25 (0.05, 1.12)	0.070	0.91 (0.20, 4.17)	0.901	2.52 (1.03, 6.19)	0.044

*Note:* OR for age at visit in this table is for ages 3 to 6. ORs for age at visit are adjusted for isoform group and GC group, ORs for isoform group comparisons are adjusted for age at visit and GC group, ORs for GC group are adjusted for age at visit and isoform group. ORs and 95% CIs are reported to two decimal places or to three decimal places when < 0.01. *p* values < 0.017 are highlighted in bold. *p* values are reported to three decimal places or < 0.001.

Abbreviations: CI, confidence interval; GC, glucocorticoids; OR, odds ratio; y, years.

### 3.4 Relationships Between Dystrophin Isoform Group and the Odds of Achieving Higher NSAA Subitem Scores

The odds of achieving a full score of 2 (compared to a score of 0 or 1) were significantly lower in Group 2 compared to Group 1 for the following 11 subitems: stand up from chair, stand on one leg—right, stand on one leg—left, climb box step—right, descend box step—right, climb box step—left, descend box step—left, lifts head, stands on heels, jump and walk run (10 m) (Table 2). Those in Group 2 had a reduction of 56% or more in the odds of achieving a full score than those in Group 1 for the following 11 subitems: stand up from chair, stand on one leg—right, stand on one leg—left, climb box step—right, descend box step—right, climb box step—left, descend box step—left, lifts head, stands on heels, jump and walk run (10 m).

The odds of achieving a full score of 2 (compared to a score of 0 or 1) were significantly lower in Group 3 compared to Group 1 for the following 5 subitems: stand on one leg—right, stand on one leg—left, descend box step—left, jump and walk run (10 m). Those in Group 3 had a reduction of 79% or more in the odds of achieving a full score than those in Group 1 for the following 5 subitems: stand on one leg—right, stand on one leg—left, descend box step—left, jump and walk run (10 m).

The odds of achieving a full score of 2 (compared to a score of 0 or 1) were significantly lower in Group 3 compared to Group 2 for stand on one leg—right and stand on one leg—left. Those in Group 3 had a reduction of 92% or more in the odds of achieving a full score than those in Group 2 for stand on one leg—right and stand on one leg—left.

**FIGURE 1:**
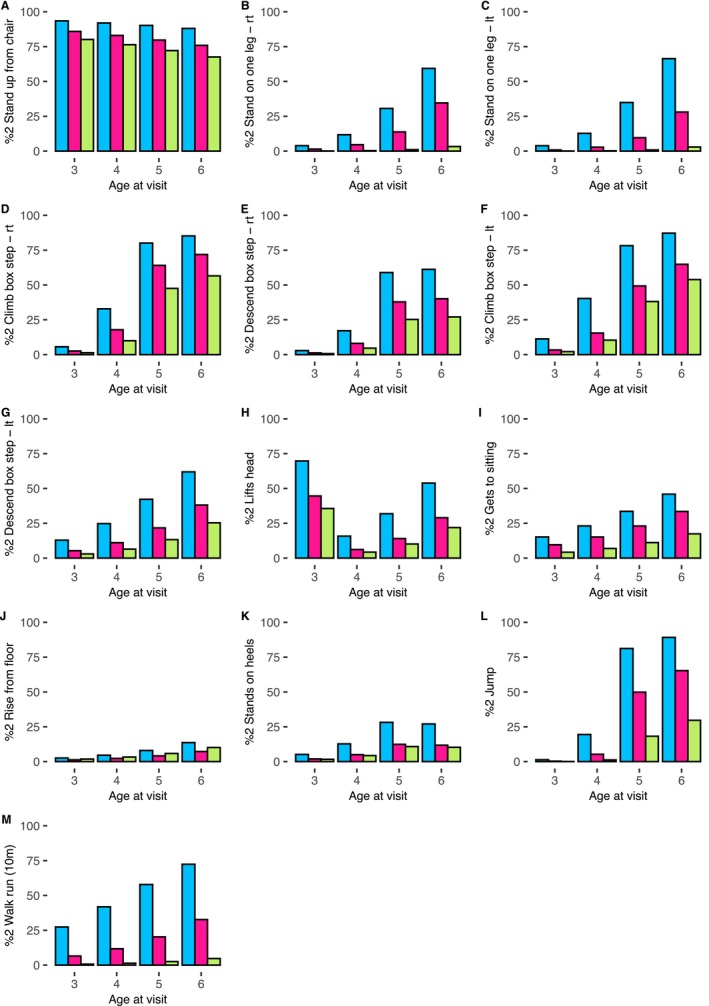
The estimated percentage achieving a full score of 2 at age 3, 4, 5 or 6 according to isoform group for those in the GC naïve group for 13 of the NSAA subitems. The 13 NSAA subitems are stand up from chair (panel A), stand on one leg ‐ right (panel B), stand on one leg ‐ left (panel C), climb box step ‐ right (panel D), descend box step ‐ right (panel E), climb box step ‐ left (panel F), descend box step ‐ left (panel G), lifts head (panel H), gets to sitting (panel I), rise from floor (panel J), stands on heels (panel K), jump (panel L) and walk run (10m) (panel M). %2 = estimated percentage achieving a full score of 2. lt, left; rt, right. Blue is isoform group 1; pink is isoform group 2 and green is isoform group 3.

The estimated percentage achieving a full score in the 13 subitems at ages 3, 4, 5 and 6 in the different isoform groups for the GC naïve group is shown in Figure 1.

Those in Group 2 had a reduction of 73% or more in the odds of achieving a full score compared to those in Group 1 for hop right leg and hop left leg (Table 3). The estimated percentage achieving a score of 2 or 1 in hop right leg and hop left leg at ages 3, 4, 5 and 6 in the different isoform groups for those in the GC naïve group is shown in the Figure S1.

### 3.5 Relationships Between GC Group and the Odds of Achieving Higher NSAA Subitem Scores

There were no significant differences in the odds of achieving higher NSAA subitem scores and GC group using a *p* value significance threshold of 0.017 after Bonferroni correction (Tables 2 and 3). Whilst we did not find any significant differences in the odds of achieving higher NSAA subitem scores and GC group, there were non‐significant trends for those in the GC treated group to have higher odds of achieving higher NSAA subitem scores compared to those in the GC naïve group.

### 3.6 Results of Sensitivity Analysis

Of the 342 included participants, 18 (5.3%) had a *DMD* mutation that was expected to be exon 44 skippable, and 5 (1.5%) had a *DMD* mutation that was a deletion of exons 3 to 7. All the participants with a deletion of exons 3 to 7 and 10 of the participants with a *DMD* mutation expected to be exon 44 skippable were in isoform Group 1. 8 of the participants with a *DMD* mutation expected to be exon 44 skippable were in isoform Group 2.

A sensitivity analysis was conducted excluding those participants with a *DMD* mutation expected to be exon 44 skippable or a deletion of exons 3 to 7. There were no changes in statistical significance for relationships between age at visit and the odds of achieving higher NSAA subitem scores apart from for lifts head for those aged 3 years (for which OR = 0.08 and *p* = 0.003 using the full dataset and OR = 0.14 and *p* = 0.027 using the sensitivity analysis dataset) and for rise from floor for those aged 3 to 6 (for which OR = 1.82 and *p* = 0.02 using the full dataset and OR = 1.95 and *p* = 0.014 using the sensitivity analysis dataset). There were no changes in statistical significance for relationships between isoform group and the odds of achieving higher NSAA subitem scores apart from when we compared the odds of achieving a full score of 2 (compared to a score of 0 or 1) for climb box step right leg in Group 2 compared to Group 1 (for which OR = 0.44 and *p* = 0.011 using the full dataset and OR = 0.49 and *p* = 0.028 using the sensitivity analysis dataset) and for descend box step left leg in Group 3 compared to Group 1 (for which OR = 0.21 and *p* = 0.015 using the full dataset and OR = 0.23 and *p* = 0.023 using the sensitivity analysis dataset). There were no changes in statistical significance for relationships between GC group and the odds of achieving higher NSAA subitem scores. There were no changes in whether the estimated OR was less than 1 or more than or equal to 1 for all estimated OR's apart from for descend box step left leg in the GC treated group compared to the GC naïve group (for which OR = 1.03 and *p* = 0.935 using the full dataset and OR = 0.95 and *p* = 0.891 using the sensitivity analysis dataset).

As the changes to statistical significance in the sensitivity analysis were minimal and only affected a small subset of the assessments of the NSAA, it was decided to include those participants with a *DMD* mutation expected to be exon 44 skippable or a deletion of exons 3 to 7 in the analysis.

## 4 Discussion

We found a strong and significant association between predicted patterns of dystrophin isoform involvement and the odds of achieving higher NSAA subitem scores, with a cumulative effect of predicted loss of isoforms. There was a strong and significant association between increasing age and higher odds of achieving higher NSAA subitem scores.

Previous studies have explored the relationship between predicted patterns of dystrophin isoform involvement and DMD central nervous system (CNS) involvement.

In a systematic review and meta‐analysis, the prevalence of intellectual developmental disorder (IDD) in DMD was 12% in participants considered to be Dp427−/Dp140+/Dp71+, 29% in participants considered to be Dp427−/Dp140−/Dp71+, and 84% in participants considered to be Dp427−/Dp140−/Dp71−, with a prevalence ratio for IDD of 0.43 for participants considered to be Dp140+/Dp71+ versus Dp140−/Dp71+ and 0.17 for Dp140+/Dp71+ versus Dp140−/Dp71− [17]. A study found that both fine and gross motor performance significantly correlated with IQ, but not with autism severity in children with ASD [18].

DMD participants with nonsense mutations downstream of exon 45 performed worse in measures of digit span, a measure of working memory, than those with mutations upstream of exon 45 [19]. A study found a higher risk of detecting difficulties in some of the Wechsler Intelligence Scale for Children (WISC‐IV) and NEPSY‐II items in the boys with certain Dp140 involvement, suggesting a specific impairment in working memory and processing speed [20]. In a study of participants with DMD, intermediate muscular dystrophy (IMD) and Becker muscular dystrophy (BMD), the Dp140− group performed worse in tests of verbal memory, executive function and attention than the Dp140+ group [4]. A meta‐analysis explored associations between different components of motor skills (balance, manual dexterity, locomotor skills and object control skills) and executive functions (response inhibition, working memory and cognitive flexibility) in typically developing children [21]. They found a positive association between executive functions (EFs) and motor skills [21]. Manual dexterity and balance were found to have the strongest independent associations with all EF components [21]. In a study of children aged 5 to 6, those with better working memory demonstrated better performance during strength, speed and manual dexterity tasks [22]. Taken together, these findings support a hypothesis that the association between expected lack of Dp140 and lower NSAA subitem scores in our study could, at least in part, be explained by deficits in executive function in those lacking Dp140.

Inattention and hyperactivity scores have been found to be higher in DMD in a Dp140− group compared with a Dp140+ group [23]. In a systematic review and meta‐analysis, in DMD, there was a reduction of 53% for the prevalence of ADHD for participants considered to be Dp71+ compared to Dp71− [24]. A systematic review reported that more than half of children with ADHD have difficulties with gross and fine motor skills [25]. This review described 3 main hypotheses to explain the impaired motor skills in children with ADHD: comorbidity, deficits in motor skills due to lack of attention, and a lack of inhibition having negative effects on motor control [25]. It may be that deficits in attention in those expected to lack Dp140 and in attention and inhibition in those expected to lack Dp71 may contribute to the worse NSAA subitem scores in our study in those expected to lack these isoforms.

An association between delay in walking and cognitive impairment in DMD has previously been described [26]. A study reported that children with DMD reported to be late walkers performed more poorly on tests of cognitive function than their on‐time peers [27].

It is possible that a lack of Dp140 has consequences for motoneuron function and this may contribute to our findings; however, further research would be needed to assess this [10].

Taken together, these findings suggest that differences in the central control of motor function in the different isoform groups may contribute to the differences in the pattern of impairment in developing gross motor skills found in the different isoform groups. However, further research would be needed to evaluate this hypothesis.

The increased use of wearable devices that passively assess patients motor performance without requiring their active collaboration [28] may help to discriminate the extent to which understanding of a given task contributes to patterns of impairment in developing gross motor skills.

Recent clinical trials have been increasingly focused on the younger DMD population and in order to interpret clinical trial results, it is important to understand the underlying drivers of the high levels of variation in DMD phenotypes in this group. Previous studies have identified delayed acquisition of motor milestones in DMD [29, 30]. This has been reflected in NSAA subitem scores explored in DMD boys aged 3 to 5 years compared to typically developing boys [31, 32]. An association between motor function and patterns of dystrophin isoform involvement has been suggested [10, 33, 34, 35, 36]. DMD boys subdivided according to expected patterns of involvement of Dp427, Dp140 and Dp71 demonstrated lower mean NSAA total scores at 5 years of age in those expected to lack Dp140 and Dp71, with a cumulative effect of loss of isoforms [10]. A study of DMD boys aged 4 to 7 reported that maximum NSAA scores were lower in those with involvement of Dp427, Dp140 and Dp71 compared to those with involvement of Dp427 and Dp140 or of Dp427 only [36]. In a study that used longitudinal latent class analysis to identify three classes of differentially progressing early age DMD motor trajectories, boys were grouped into dystrophin isoform groups as follows: Dp427 absent and Dp140/Dp71 present (Group 1); Dp427/Dp140 absent and Dp71 present (Group 2); and Dp427/Dp140/Dp71 absent (Group 3) [34]. Dystrophin isoform groupings and clustering of longitudinal trajectories were associated with 56.5% of those from Group 1, 44.5% of those from Group 2, and 22.2% of those from Group 3 being in the slowest progressing class [34]. 133 DMD boys aged 1.5 to 18 years, with a median age at baseline of 7 years, were grouped into Group 1 (predicted to lack Dp116, Dp140 and Dp71) and Group 2 (Dp116, Dp140 and Dp71 predicted to be not affected) [35]. They reported an accelerated decline in hip and knee strength and the NSAA activities standing on one leg right and left and walk, as well as an earlier age of becoming wheelchair bound in Group 1 compared to Group 2 [35]. In a study of GC naïve DMD boys aged 4 to under 7 years, participants with distal *DMD* mutations (mutations in 3′ *DMD* including intron 44) had lower 10‐m walk/run velocity and rise from floor velocity than those with proximal *DMD* mutations (proximal to intron 44) [33].

Currently, DMD clinical trials do not typically stratify participants according to expected patterns of dystrophin isoform involvement. The NSAA is commonly used as an outcome measure in clinical trials. Our results demonstrate that expected patterns of dystrophin isoform involvement are an important factor to consider when evaluating differences in NSAA scores, and if these are not accounted for during clinical trial design, this could compromise the demonstration of a treatment effect and the understanding of the results of clinical trials.

The consistency of data derived from both the North Star Clinical Network and the AFM funded study with data derived from other data sources, including populations from Europe and the United States of America, has previously been reported [37, 38, 39].

Limitations of our study include the smaller sample size for Group 3, in keeping with the naturally occurring frequency of these mutations, and hence the large confidence intervals for the estimates relating to Group 3, as well as missing data. We note that for all the NSAA subitems studied apart from rise from floor, the odds of achieving higher NSAA subitem scores was lower for Group 3 compared to Group 2. However, these differences only reached statistical significance, after Bonferroni correction, for stand on one leg right and left. This may be in part related to the smaller sample size in Group 3, and it may be that if a larger number of participants from Group 3 were able to be studied, it may be possible to demonstrate stronger statistical significance. Those in the GC naïve group were those who had not been on GC for 6 months or more at the first visit, and as such, this group did not consist of participants who had all been completely unexposed to GC. In addition, other *DMD* mutation types and changes in genes other than *DMD* (gene modifiers) that were not assessed in this study could also contribute to changes in the outcomes studied.

In conclusion, in our study we found a strong association between predicted lack of Dp140 and deficits in aspects of the gross motor developmental subitems tested in the NSAA. A cumulative effect of the additional lack of Dp71 was found for standing on one leg bilaterally. This gives important insight into the potential role of Dp140 and Dp71 in not only cognitive function, but also the higher aspects of the control of balance and motor function in DMD.

## Author Contributions

M.C., D.R., G.S., L.S., A.M., the UK NorthStar Clinical Network and F.M. contributed to the conception of the study. M.C., D.R., G.S., L.S., G.B., A.M., the UK NorthStar Clinical Network and F.M. contributed to the design of the study. M.C. drafted the manuscript and performed the statistical analysis. M.C., D.R., G.S., L.S., G.B., A.M., the UK NorthStar Clinical Network and F.M. contributed to the editing of the manuscript.

## Conflicts of Interest

Mary Chesshyre, Deborah Ridout, Georgia Stimpson and Adnan Manzur report no financial disclosures or potential conflicts of interest. Laurent Servais reports consultancy/board member in DMD for Dyne, Pepgen, Santhera, Pfizer, Roche, Santhera and Italfarmaco. Laurent Servais has received personal fees from Dyne, Pepgen, Wave, Roche, Italfarmaco, Santhera and Pfizer. Francesco Muntoni reports grants for the submitted work from Muscular Dystrophy UK (Grant reference: 22GRO‐PG24‐0598) and Brain Involvement iN Dystrophinopathies (BIND) European Union Horizon 2020 award Grant agreement: 847826. The BIND project (grant agreement No: 847826 [40]) has received funding from the European Union's Horizon 2020 research and innovation programme. Outside of the submitted work, Francesco Muntoni has received a grant from Sarepta Therapeutics (D‐BRAIN grant to UCL) and personal fees from Dyne Therapeutics (SAB), Sarepta (SAB) and PTC Therapeutics (SAB). Giovanni Baranello reports grants and personal fees from Sarepta and Roche and personal fees from Pfizer from outside of the submitted work.

## Supporting information


Figure S1. The estimated percentage achieving a score of 2 or 1 at age 3, 4, 5 or 6 for hop right leg (panel A) and hop left leg (panel B) according to isoform group for those in the GC naïve group. %2 or 1 = estimated percentage achieving a subitem score of 2 or 1. Blue is isoform group 1; pink is isoform group 2 and green is isoform group 3.

## Data Availability

The data that support the findings of this study are available from the corresponding author (Francesco Muntoni) upon reasonable request.

## Appendix 1

### Additional Information About Participants' GC Medication and Regime

For the 66 participants in the ‘GC treated’ group, 34 were recorded as having prednisolone, 14 were recorded as having deflazacort, 1 was recorded as having prednisolone or deflazacort (different medication recorded at different visits) and for 17 participants, the type of GC medication (prednisolone or deflazacort) was missing. For the 66 participants in the ‘GC treated’ group, the type of GC medication was missing for 51 visits. For the 66 participants in the ‘GC treated’ group, 20 were recorded as being on a ‘daily’ regime, 15 on an ‘intermittent’ regime, 6 on an ‘other’ regime, 3 on a ‘daily’ or ‘intermittent’ regime (different regimes recorded at different visits), 2 on an ‘every other day’ or ‘alternate days’ regime, 1 was recorded as on a ‘daily’ or ‘other’ regime (different regimes recorded at different visits) and the regime was missing for 19 participants. For the 66 participants in the ‘GC treated’ group, the GC regime was missing for 52 visits.
