# Supplementary figures and images for: Influence of Dystrophin Isoform Deficiency on Motor Development in Duchenne Muscular Dystrophy
Source: Ann Clin Transl Neurol. 2025 Jun 24;12(9):1732–42. doi: 10.1002/acn3.70097 (PMC12455871; doi:10.1002/acn3.70097)

**A**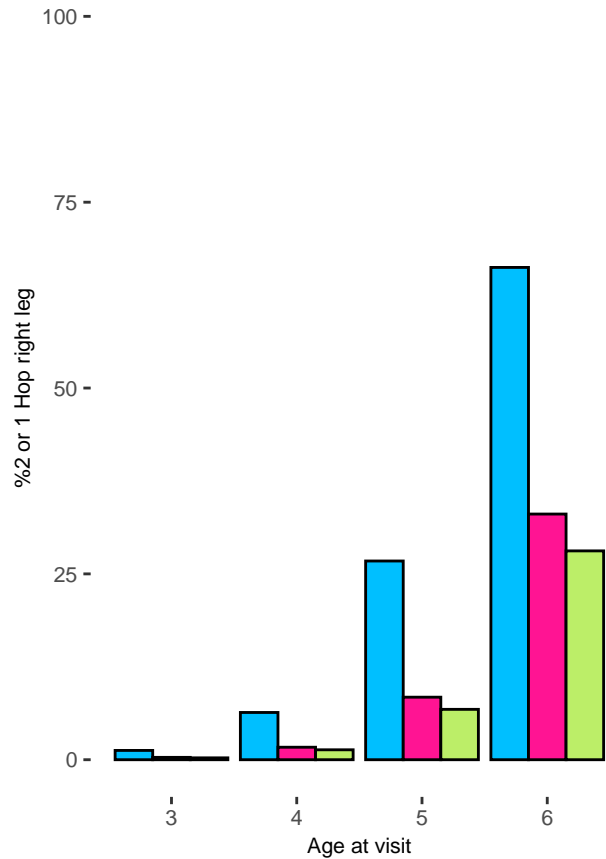**B**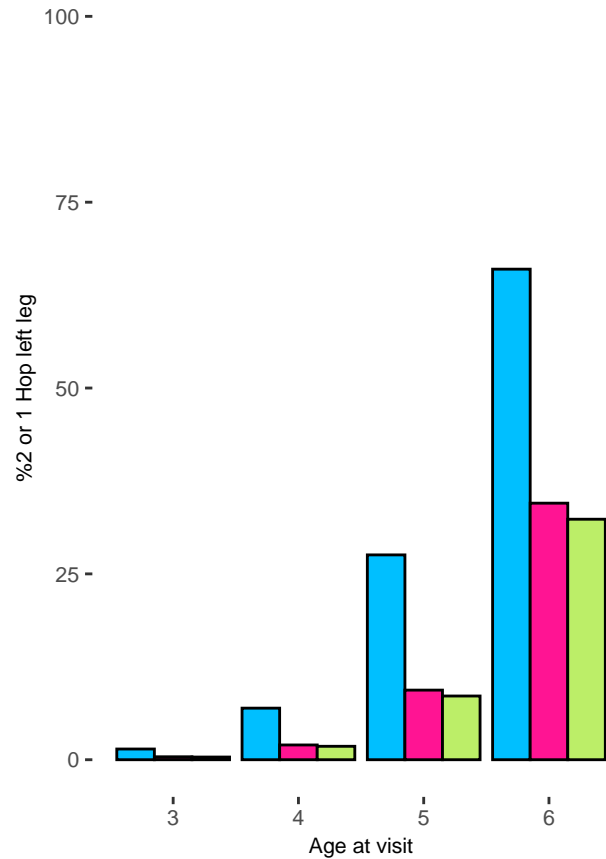

Supplement: Supplementary file 1 — Figure S1. The estimated percentage achieving a score of 2 or 1 at age 3, 4, 5 or 6 for hop right leg (panel A) and hop left leg (panel B) according to isoform group for those in the GC naïve group. %2 or 1 = estimated percentage achieving a subitem score of 2 or 1. Blue is isoform group 1; pink is isoform group 2 and green is isoform group 3. [file ACN3-12-1732-s001.pdf]
